# A cluster of palmitoylated cysteines are essential for aggregation of cysteine-string protein mutants that cause neuronal ceroid lipofuscinosis

**DOI:** 10.1038/s41598-017-00036-8

**Published:** 2017-01-31

**Authors:** Cinta Diez-Ardanuy, Jennifer Greaves, Kevin R. Munro, Nicholas C. O. Tomkinson, Luke H. Chamberlain

**Affiliations:** 1 Strathclyde Institute of Pharmacy & Biomedical Sciences, University of Strathclyde, 161 Cathedral Street, Glasgow, G4 0RE UK; 2 WestCHEM, Department of Pure and Applied Chemistry, University of Strathclyde, 295 Cathedral Street, Glasgow, G1 1XL UK

## Abstract

Autosomal-dominant adult-onset neuronal ceroid lipofuscinosis (ANCL) is caused by mutation of the *DNAJC5* gene encoding cysteine string protein alpha (CSPα). The disease-causing mutations, which result in substitution of leucine-115 with an arginine (L115R) or deletion of the neighbouring leucine-116 (∆L116) in the cysteine-string domain cause CSPα to form high molecular weight SDS-resistant aggregates, which are also present in post-mortem brain tissue from patients. Formation and stability of these mutant aggregates is linked to palmitoylation of the cysteine-string domain, however the regions of the mutant proteins that drive aggregation have not been determined. The importance of specific residues in the cysteine-string domain was investigated, revealing that a central core of palmitoylated cysteines is essential for aggregation of ANCL CSPα mutants. Interestingly, palmitoylated monomers of ANCL CSPα mutants were shown to be short-lived compared with wild-type CSPα, suggesting that the mutants either have a faster rate of depalmitoylation or that they are consumed in a time-dependent manner into high molecular weight aggregates. These findings provide new insight into the features of CSPα that promote aggregation in the presence of L115R/∆L116 mutations and reveal a change in the lifetime of palmitoylated monomers of the mutant proteins.

## Introduction

A mechanistic link between protein aggregation and neurodegeneration is considered to be well established in several disorders, such as Alzheimer’s disease, Parkinson’s disease and Huntington’s disease^1–3^. Two mutations in the *DNAJC5* gene encoding CSPα, which result in a substitution of leucine-115 by arginine (L115R) or a deletion of leucine-116 (ΔL116), have been identified as the cause of the neurodegenerative disorder adult-onset neuronal ceroid lipofuscinosis (ANCL)^4,5^. We previously reported that these mutations cause CSPα to form high molecular weight SDS-resistant aggregates^6^, suggesting that protein aggregation may also be associated with neurodegeneration in ANCL. Indeed, SDS-resistant CSP aggregates were detected in post-mortem brain tissue from individuals carrying the L115R mutation^6^.

The amino acid changes (L115R and ΔL116) that occur as a result of the disease-causing mutations are located within the cysteine-string domain (CSD), a region of the protein that is highly modified by palmitoylation of up to 14 densely-packed cysteine residues^7,8^. Our previous work showed that aggregation of the ANCL mutants was linked to palmitoylation as it was enhanced by co-expression of active (but not inactive) “zDHHC” palmitoyltransferase enzymes and was reduced by hydroxylamine treatment, which depalmitoylates CSPα^6^. Post-translational modifications have been shown to impact protein aggregation in other neurodegenerative disorders, such as Huntington’s disease, Parkinson’s disease and Alzheimer’s disease^9–11^. Indeed, palmitoylation has previously been implicated in neurodegeneration as the formation of inclusions containing mutant huntingtin is increased when palmitoylation of the protein is blocked^12^.

However, an increased aggregation of the ANCL CSPα mutants compared with wild-type protein was also seen with bacterially-produced recombinant proteins, which lack palmitoyl modifications^13^, although it is unclear if these aggregates/oligomers are the same as those formed from palmitoylated proteins in cells. Indeed, differences in the oligomerisation properties of non-palmitoylated and palmitoylated wild-type CSPα have previously been documented^14^. Intriguingly, levels of the lysosomal thioesterase enzyme PPT1 (which removes acyl chains from palmitoylated proteins during their degradation) were recently shown to be markedly increased in brain samples from ANCL patients^15^, further supporting a link between palmitoylation and ANCL. Indeed, we previously proposed that palmitoylated ANCL CSPα mutants present within aggregates may be inaccessible to PPT1 and that the resulting deficit in degradative protein depalmitoylation could be the trigger for this lysosomal-storage disorder^6^. Intriguingly, mutations in PPT1 that block activity or lysosomal targeting cause early-onset forms of NCL^16^, further suggesting that deficits in the turnover of palmitoylated proteins could lead to lysosomal dysfunction.

In order to develop therapeutic strategies to treat NCL, it is important to identify pathways and mechanisms that lead to pathogenesis. We have proposed that CSPα aggregation is the trigger for neurodegeneration in ANCL and have therefore investigated the features of ANCL CSPα mutants that mediate this aggregation. Given the previous identified links between palmitoylation and aggregation^6^, this study has focused on the importance of specific cysteines in the CSD for aggregation.

## Results

### Analysis of the effects of cysteine substitutions on aggregation of the L115R and ΔL116 CSPα mutants

**Figure 1:**
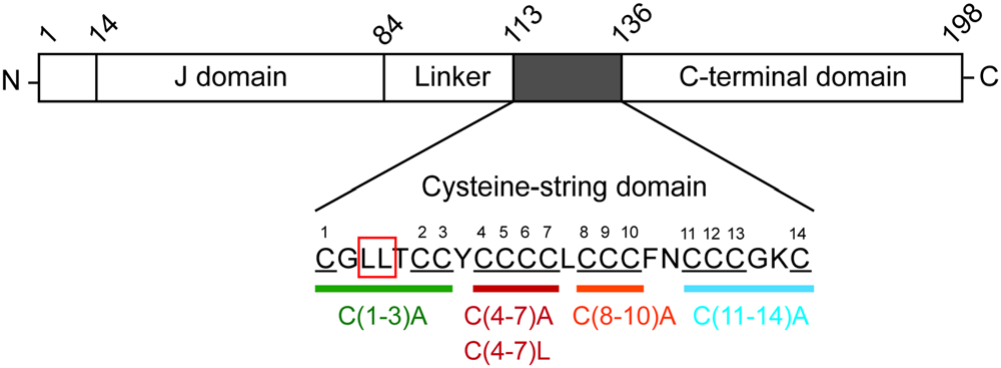
Schematic of the cysteine substitutions introduced into the cysteine-string domain. Schematic diagram of CSPα showing relative positions of the different domains of the protein and highlighting the positions of amino acids L115R and L116 within the cysteine-string domain (CSD). The cysteines present in the CSD are numbered from 1 to 14 and the different cysteine substitution mutants that were generated are indicated in different colours.

Our previous study showed that aggregation of ANCL CSPα mutants is closely associated with palmitoylation. Specifically, we found that: (i) the presence of SDS-resistant aggregates was reduced following treatment with hydroxylamine; and (ii) co-expression of active (but not inactive) zDHHC enzymes stimulated increased aggregation of the ANCL mutants^6^. To explore the aggregation process further, we have examined how specific palmitoylated cysteines contribute to this process by generating and analysing a panel of ANCL CSPα mutants bearing specific cysteine-to-alanine substitutions (see Fig. 1 for schematic diagram).

Cysteine substitutions were introduced into EGFP-tagged CSPα constructs (as per our previous work ref. 17) and also into untagged CSPα constructs to exclude any confounding effects of the EGFP tag on the results that were obtained.

**Figure 2:**
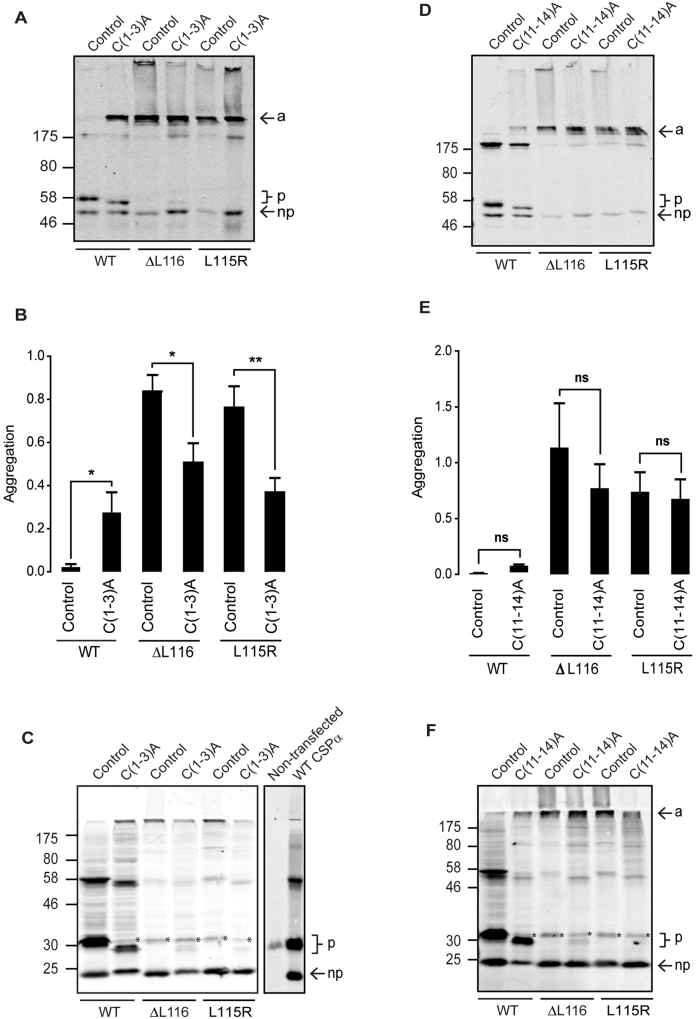
Effect of C(1-3)A and C(11-14)A substitutions on aggregation of CSPα ANCL mutants. PC12 cells were transfected for 48 hours with EGFP-tagged or untagged CSPα constructs. Cell lysates were resolved by SDS-PAGE and analysed by immunoblotting with anti-GFP antibody (**A** and **D**) and anti-CSPα antibody (**C** and **F**). *a* indicates aggregates, *p* shows position of palmitoylated monomeric CSPα, and *np* designates the non-palmitoylated monomers. Position of size markers are shown on the left hand side. Asterisks in panels C and F indicate endogenous palmitoylated CSPα. In panel C, the immunoblot on the right provides a comparison of CSPα expression levels in untransfected and transfected cells. Quantification of EGFP-CSPα proteins is presented as a ratio of aggregated to monomeric + dimeric forms of the proteins (n = 4), shown together with SEM (error bars) (panels B and E). The data was analysed using an unpaired Student’s T test; asterisks denote a significant difference (*p < *0.05*, **p < *0.01*) from the respective control CSPα construct (wild-type, ΔL116 or L115R).

The effects of cysteine substitutions at either end of the CSD in wild-type, L115R and ∆L116 proteins are presented first. Previous work showed that C(1-3)A and C(11-14)A mutants are membrane-associated and efficiently palmitoylated on the remaining cysteines^17^. Figure 2 presents representative immunoblots of wild-type, L115R and ∆L116 proteins containing C(1-3)A and C(11-14)A substitutions expressed in PC12 cells, and corresponding quantification of the ratio of aggregates to monomeric plus dimeric forms of the protein (which corresponds to the sum of monomeric non-palmitoylated and palmitoylated bands, and the putative dimeric band at ~175 kDa). As can be seen, cysteine substitutions at positions 1-3 led to a faster migration on SDS gels of the palmitoylated protein compared to the wild-type palmitoylated protein, consistent with the reduced number of palmitoylation sites (Fig. 2A and C). However, in agreement with previous work^17^, there was no obvious change in the efficiency of palmitoylation of the remaining cysteines in wild-type CSPα carrying C(1-3)A substitutions (Fig. 2A and C). Introducing the C(1-3)A substitutions into the ANCL mutants did not have a major effect on their migration profile (Fig. 2A–C). Although there was a significant reduction in the level of aggregation of the ANCL mutants containing the C(1-3)A substitutions (Fig. 2B), there was only a very small amount of palmitoylated monomeric ΔL116 and L115R mutants observed and substantially less than seen with the C(1-3)A-substituted wild-type CSPα (Fig. 2A and C). As can be seen in Fig. 2C, the expression levels of exogenous untagged CSP were several times higher than the endogenous protein in PC12 cells, and thus endogenous CSPα has only a very minimal contribution to the overall immunoreactive signal of transfected cells.

Similar to the C(1-3)A substitutions, introduction of the C(11-14)A substitutions into wild-type CSPα did not affect the overall palmitoylation efficiency of the remaining cysteines, although the palmitoylated band migrated faster, consistent with the removal of a number of palmitoylation sites (Fig. 2D and F). As can be seen from Fig. 2D–F, substitution of the cysteines at positions 11-14 did not have any major effect on the migration profile of the EGFP-tagged or untagged CSPα ANCL mutants, suggesting that these residues are not central to the aggregation process.

**Figure 3:**
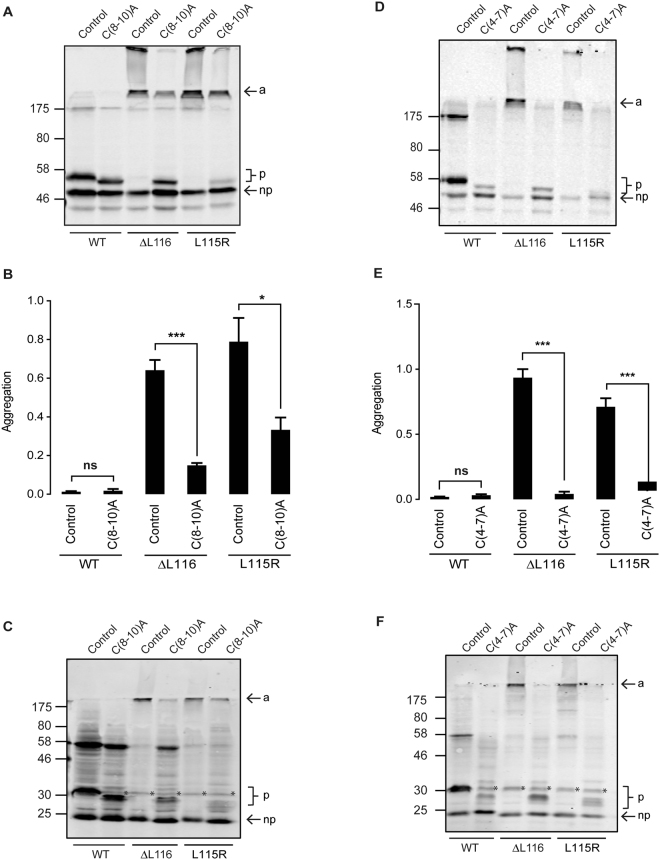
Effect of C(8-10)A and C(4-7)A substitutions on aggregation of CSPα ANCL mutants. PC12 cells were transfected for 48 hours with EGFP-tagged or untagged CSPα constructs. Cell lysates were resolved by SDS-PAGE and analysed by immunoblotting with anti-GFP antibody (**A** and **D**) and anti-CSPα antibody (**C** and **F**). *a* indicates aggregates, *p* shows position of palmitoylated monomeric CSPα, and *np* designates the non-palmitoylated monomers. Position of size markers are shown on the left hand side. Asterisks in panels C and F indicate endogenous palmitoylated CSPα. Quantification of EGFP-CSPα proteins is presented as a ratio of aggregated to monomeric + dimeric forms of the proteins (n = 4), shown together with SEM (error bars) (panels B and E). The data was analysed using an unpaired Student’s T test; asterisks denote a significant difference (*p < *0.05*, ***p < *0.001*) from the respective control CSPα construct (wild-type, ΔL116 or L115R).

In contrast to the cysteine residues that flank the CSD, substitution of cysteines in the core of this domain had a major effect on aggregation of the ANCL mutants. As with the other cysteine mutants studied, substitution of cysteines 8-10 to alanines led to a band-shift in wild-type CSPα, consistent with removal of palmitoylated residues but did not affect the efficiency of palmitoylation of the remaining cysteines (Fig. 3A and C). Substitution of these cysteines had a clear effect on the ANCL mutants by reducing their aggregation and also increasing levels of a monomeric palmitoylated band, which was more prominent with the ∆L116 mutant than the L115R mutant (Fig. 3A and C). However, despite the reduction in aggregation of the ANCL mutants when these cysteine residues were substituted, aggregation was not abolished (Fig. 3A–C).

**Figure 4:**
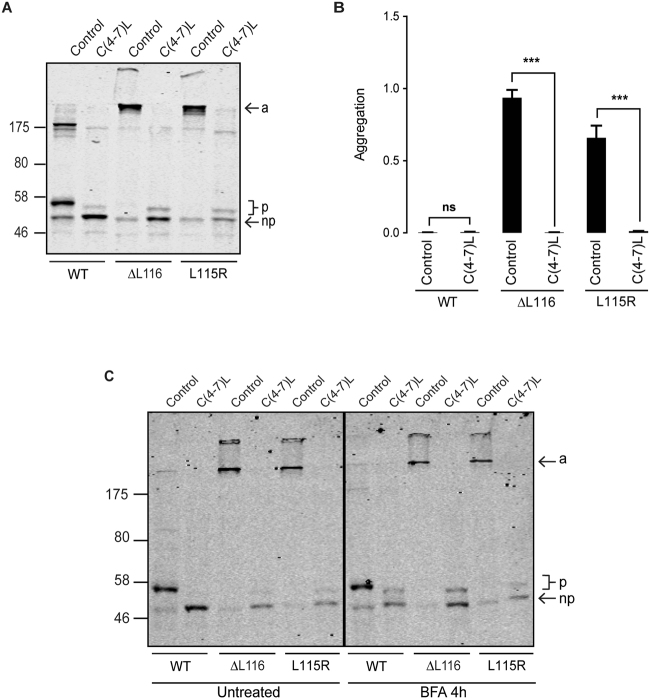
C(4-7)L substitutions block aggregation of the ANCL mutants. EGFP-CSPα wild-type and ΔL116/L115R constructs with or without C(4-7)L substitutions were transfected into PC12 cells for 48 hours and subsequently analysed by immunoblotting with anti-GFP (**A**). The non-palmitoylated monomers (*np*), palmitoylated monomers (*p*) and aggregates (*a*) are marked by arrows. The position of molecular marker is shown on the left hand side. Averaged data of the aggregate to monomer + dimer ratio (n = 4) is shown together with SEM (**B**). Statistical significance was assessed using an unpaired Student T test, asterisks denote a significant difference compared with the control (wild-type, ΔL116 or L115R) CSPα construct (***p < *0.001*). (**C**) PC12 cells transfected with the wild-type and mutant EGFP-CSPα constructs with or without the C(4-7)L substitutions were treated with 30 µg/ml of BFA for 4 hours. The samples were then resolved by SDS-PAGE and transferred to nitrocellulose for immunoblotting analysis using an antibody against GFP. The untreated and BFA-treated samples that are shown are from the same immunoblot but with intervening gel lanes removed and this is indicated by the solid black line.

As previously reported, replacement of the cysteine residues at positions 4-7 of the CSD with alanines decreased the overall efficiency of palmitoylation of the remaining cysteines in wild-type CSPα (Fig. 3D and F; compare immunoreactivity of palmitoylated and non-palmitoylated monomeric bands of wild-type CSPα and C(4-7)A)^17^. This is thought to reflect a role for this cluster of cysteines (and their hydrophobicity) in membrane association prior to palmitoylation. Interestingly, substitution of these cysteines caused a complete loss of aggregation and recovered the palmitoylated monomeric band of the ANCL mutants to a similar level as seen for wild-type CSPα bearing the C(4-7)A substitutions (Fig. 3D–F). The palmitoylation band shift was smaller for the EGFP-tagged L115R mutant with C(4-7)A substitutions (Fig. 3D) and more diffuse for the untagged version of this protein (Fig. 3F), suggesting that this ANCL mutant may have an underlying disruption in palmitoylation that is uncovered when its aggregation is prevented. However, an issue to consider is that wild-type CSPα with C(4-7)A substitutions is not efficiently palmitoylated on its remaining cysteines and is more cytosolic than wild-type CSPα^17^. Thus, it is possible that loss of aggregation of the ANCL mutants carrying C(4-7)A substitutions is due to reduced membrane association rather than a specific role of these cysteines in the aggregation process. We previously showed that membrane association (but not palmitoylation) could be preserved when cysteines 4-7 are replaced by more hydrophobic leucine residues^17^. CSPα carrying C(4-7)L substitutions associates tightly with membranes but localises to the ER, which prevents its palmitoylation due to physical separation from its partner zDHHC enzymes, which are Golgi-localised^18^. Therefore, to determine if the loss of aggregation of the ANCL CSPα mutants with C(4-7)A substitutions was simply due to a loss of membrane association, we also examined the effects of C(4-7)L substitutions. Consistent with previous work, only a very faint band representing palmitoylated C(4-7)L protein was detected and the bulk of this protein was non-palmitoylated (Fig. 4A). Importantly, introducing cysteine to leucine substitutions at these positions in the ANCL mutants led to a complete loss of aggregation (Fig. 4A and B) similar to that seen with the C(4-7)A mutants, further emphasising the importance of these cysteines (rather than membrane association) for aggregation of ANCL CSPα mutants.

### Driving palmitoylation of the C(4-7)L ANCL mutants with Brefeldin A does not promote their aggregation

Although C(4-7)A and C(4-7)L substitutions reduce aggregation of the ANCL mutants, these amino acid substitutions perturb palmitoylation of the remaining cysteines in the CSD. It was therefore important to rule out the possibility that loss of aggregation was due to a block of palmitoylation of the entire CSD. It has previously been shown that addition of brefeldin A (BFA) to cells expressing CSPα with C(4-7)L substitutions results in palmitoylation of this protein due to mixing of ER and Golgi membranes, where the C(4-7)L protein and zDHHC3/7/17 are localised, respectively^18^. Therefore, in order to promote palmitoylation of the CSPα proteins carrying C(4-7)L substitutions, transfected PC12 cells were treated with BFA for 4 hours. Although BFA induced palmitoylation of the proteins carrying C(4-7)L substitutions, this did not lead to aggregation of the ANCL CSPα mutants (Fig. 4C). These results re-emphasize the direct role that cysteines 4-7 are likely to play in the formation of ANCL mutant CSPα aggregates.

### Analysis of the effects of zDHHC enzyme co-expression on aggregation of ANCL mutant CSPα

**Figure 5:**
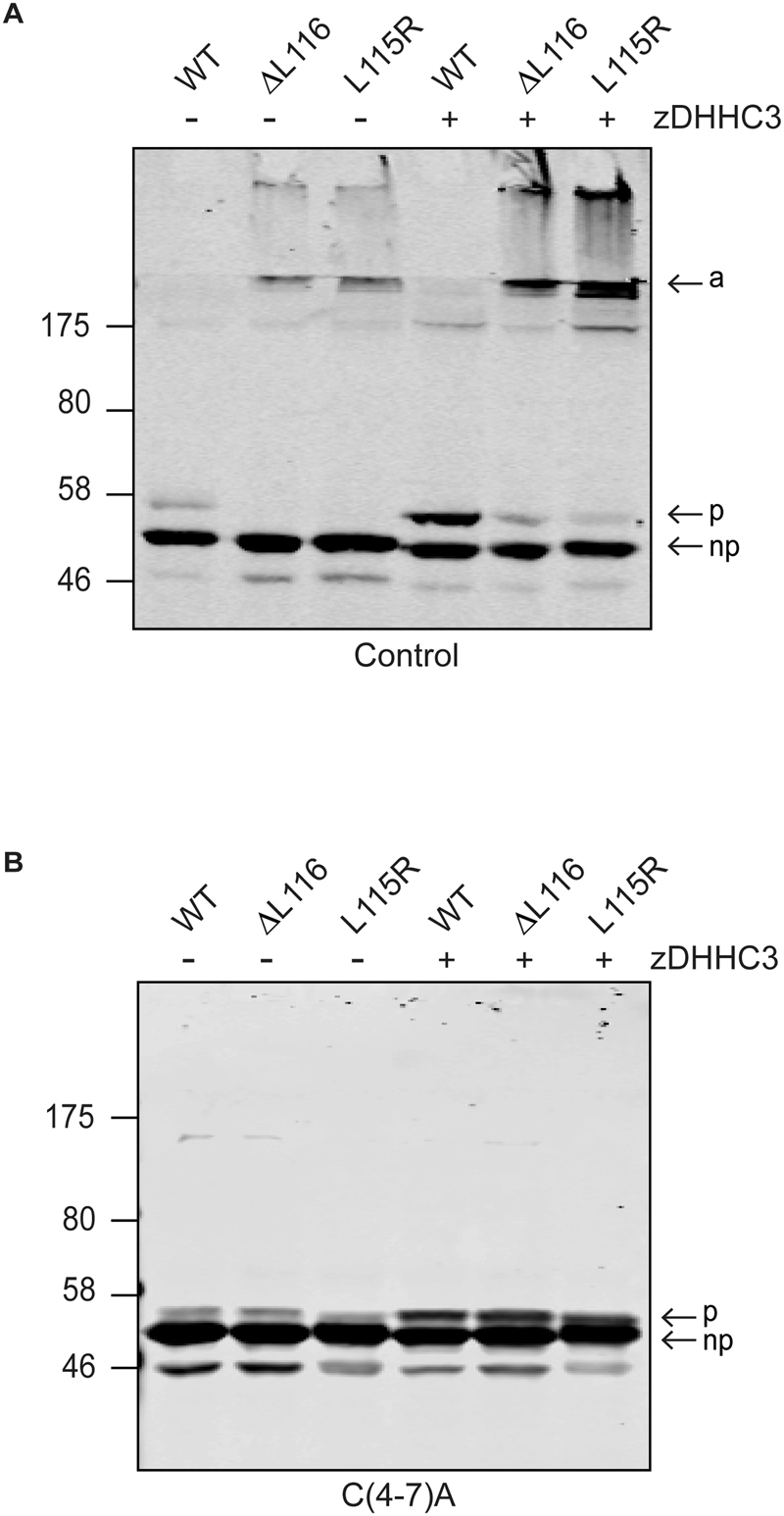
Effect of palmitoylation on aggregation of ANCL mutants. HEK293T cells were transfected for 24 hours with wild-type, L115R or ΔL116 EGFP-CSPα constructs, without (**A**) or with (**B**) the C(4-7)A substitutions, and with or without HA-zDHHC3 as indicated. Cells were then lysed and examined by immnoblotting with anti-GFP. The position of the molecular weight marker is shown on the *left*, and the arrows indicate the aggregated (*a*), palmitoylated monomeric (*p*) and non-palmitoylated monomeric (*np*) forms of the protein.

Our previous work showed that co-expression of active zDHHC enzymes (−3, −7 or −17) led to increased aggregation of ANCL CSPα mutants^6^, consistent with a role for palmitoylation in the aggregation process. However, it is important to ensure that this aggregation of ANCL mutants is not caused by some indirect (palmitoylation-independent) effect of zDHHC enzyme over-expression^13^. To investigate this issue, HEK293T cells were co-transfected with HA-zDHHC3 and wild-type or ANCL mutant CSPα with or without C(4-7)A substitutions. As expected, palmitoylation of wild-type CSPα was increased when co-expressed with zDHHC3 (Fig. 5A). Moreover, aggregation of the ANCL mutants was also enhanced by zDHHC3 co-expression, as previously reported^6^ (Fig. 5A). However, no formation of high molecular weight SDS-resistant aggregates was detected for ANCL CSPα mutants carrying the C(4-7)A substitutions when co-expressed with zDHHC3 (Fig. 5B). This result suggests that over-expression of zDHHC enzymes does not induce aggregation of ANCL mutants *via* some indirect effect but instead that aggregation is *directly* linked to increased palmitoylation of the mutants. This analysis further emphasises the importance of cysteines 4-7 for ANCL mutant CSPα aggregation.

### Palmitoylated monomers of ANCL CSPα mutants are more short-lived than palmitoylated monomers of wild-type CSPα

**Figure 6:**
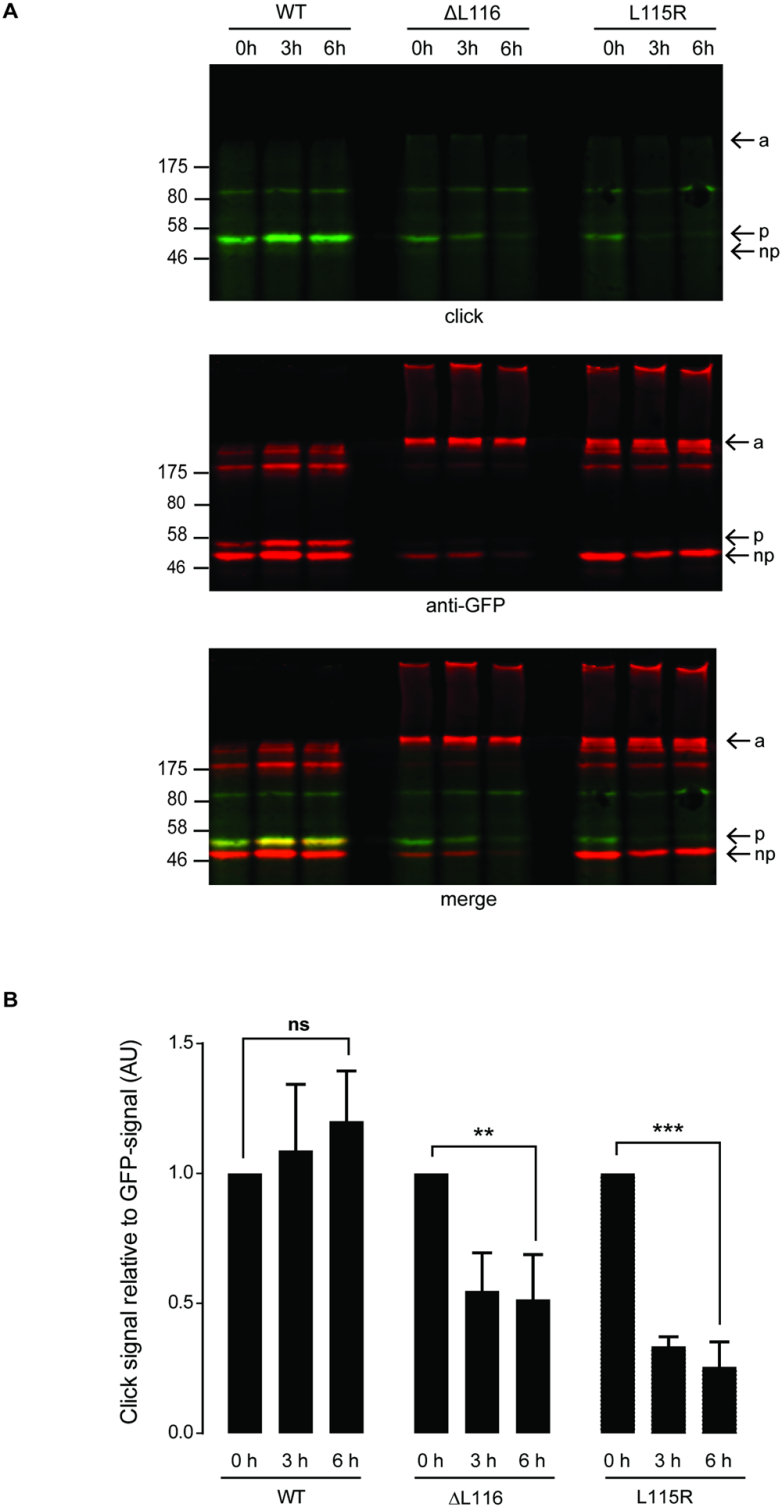
Palmitoylated monomers of ANCL CSPα mutants are more short-lived than wild-type protein. HEK293T cells were co-transfected with HA-zDHHC3 and wild-type, ΔL116 or L115R EGFP-CSPα for 24 hours as indicated. Cells were then labelled with 100 μM palmitic acid azide for 4 hours. After the metabolic labelling, cells were incubated with 100 μM unlabelled palmitic acid and 50 µM of cycloheximide for 3 to 6 hours as indicated. Following lysis, incorporation of palmitic acid azide was detected by click reaction with IRDye 800CW alkyne. Precipitated proteins were resolved by SDS-PAGE and analysed by immunoblotting. (**A**) Representative images showing click signal from wild-type, ΔL116 and L115R CSPα (*top panel*), GFP immunoreactivity (*middle panel*) and a merge (*bottom panel*). Position of molecular weight markers are shown on the left, and the arrows indicate the aggregated (*a*), palmitoylated monomeric (*p*) and non-palmitoylated monomeric (*np*) forms of the proteins. (**B**) The graph shows quantification of palmitic acid azide incorporation into the EGFP-CSPα constructs. Quantification was performed by densitometry and is expressed as a ratio of click signal to anti-GFP signal. The calculated values of palmitic acid azide incorporation into the EGFP-CSPα constructs at 0 h were normalised to 1 for each construct and the chase values were expressed relative to this. Statistical analyses were completed with one-way ANOVA (n = 3), asterisks denote a significant difference (ns = *non-significant*, **p < *0.01*, ***p < *0.001*).

Given the links between palmitoylation and aggregation of the ANCL CSPα mutants^6^ and our demonstration here that palmitoylated cysteines in the core of CSD are key to the aggregation process, we subsequently examined if there are differences in turnover of palmitoylation in wild-type CSPα and ANCL CSPα mutants. For this, HEK293T cells were used as higher protein expression levels allow for more sensitive detection of palmitoylation. Cells expressing EGFP-tagged CSPα and HA-zDHHC3 were labelled with palmitic acid azide for four hours. The label was then removed and replaced with media containing cycloheximide to block new protein synthesis for either 3 or 6 hours. Cell lysates were then incubated with an infrared dye conjugated to an alkyne group, which fluorescently labelled palmitoylated proteins by a click reaction. Figure 6 shows that there was no loss of click signal for wild-type CSPα even after a 6 hour chase period, consistent with the protein being stably palmitoylated. In contrast, there was a marked and significant loss of click signal on palmitoylated monomers of the ANCL CSPα mutants. This suggests either that the palmitoylated mutant monomers have a faster rate of depalmitoylation than wild-type CSPα or that the palmitoylated monomers are being consumed into high molecular weight aggregates in a time-dependent manner. Labelling of the ANCL aggregates with alkyne dye was quite low. This may reflect a relative inaccessibility of azide groups within tightly-packed aggregates or that monomers within the aggregates are palmitoylated at a lower level than non-aggregated monomers. There was no obvious time-dependent increase in labelling of the ANCL aggregates, which could also be linked to inefficiency of click-based detection, specifically in the context of aggregated proteins.

## Discussion

The results of this study implicate specific cysteines in the core of the CSD in the formation of high molecular weight SDS-resistant aggregates by the ANCL CSPα mutants. It is clear that these cysteines in wild-type CSPα are usually modified by palmitoylation as their alanine substitution leads to a marked band-shift on SDS gels, consistent with a loss of palmitoylation. CSPα is unique in the level of its palmitoylation and density of cysteine residues in the CSD. This may make the protein prone to aggregation^14^, particularly when mutations in surrounding residues (i.e. L115/L116) disrupt the normal sequence/structure of this domain. Indeed, wild-type CSPα forms dimers in cells (e.g. reference 19) and bacterially-produced non-palmitoylated CSPα forms higher molecular weight oligomers^14^, emphasising the intrinsic tendency of the protein to self-associate.

Although substitution of cysteines at positions 4-7 was most effective at preventing aggregation of ANCL CSPα mutants, substitution of the adjacent cysteines (8-10) also had a marked effect on aggregation and on the presence of palmitoylated monomeric CSPα on SDS gels. Thus, it is likely that SDS-resistant aggregates are formed/stabilised by palmitoylated cysteines in the central core of the CSD (i.e. Cys 4-10). ANCL mutations may alter the structure of the CSD in such a way that hydrophobic palmitoyl chains in the centre of the CSD no longer undergo optimal membrane interactions, leading to aggregation *via* hydrophobic interactions. Alternatively, the ANCL mutations could lead to a change in palmitoylation of the CSD, for example, by preventing palmitoylation of certain cysteine residues. If palmitoylation of the CSD is important for the overall structure of this domain then inefficient palmitoylation may also be a factor leading to aggregation. It is interesting to note that although the C(8-10)A substitutions reduced aggregation of the L115R mutant and recovered a palmitoylated monomeric band on SDS gels, the size of this band is consistent with inefficient palmitoylation of the remaining cysteines compared to wild-type CSPα.

Previous research by our group^17^ has shown that whereas alanine substitution of C(1-3), C(8-10) and C(11-14) does not affect palmitoylation of the remaining cysteines in the CSD, substitution of C(4-7) does. The essential role of cysteines 4-7 for efficient palmitoylation of the CSD appears to relate to a requirement of these cysteines (and in particular their hydrophobic character) for initial membrane binding of CSPα prior to palmitoylation^18^. However, loss of membrane association could not account for abolished aggregation of the ANCL CSPα mutants carrying C(4-7)A substitutions as leucine replacement of these cysteines (which allows tight membrane association) also prevented aggregation. Another possibility was that substitution of cysteines at positions 4-7 prevented aggregation because of a loss of palmitoylation of the remaining cysteines in the CSD rather than just a loss of cysteines 4-7. To address this, BFA was used to fuse ER and Golgi membranes in cells expressing the ANCL CSPα mutants with C(4-7)L substitutions^18^. Although this led to an increase in palmitoylation, it did not promote aggregation of the ANCL CSPα mutants lacking cysteines 4-7, clearly suggesting that these cysteines are integral to the aggregation process.

Although substitution of cysteines 1-3 reduced aggregation, it was difficult to interpret these results as substantial aggregation of the ANCL mutants was still observed and substitution of these cysteines (which surround residues L115 and L116), also promoted the formation of aggregates in the wild-type protein. However, it was clear that although aggregation of the ANCL mutants was partially reduced by the C(1-3)A substitutions, there was very little rescue of a palmitoylated monomeric form of the protein on SDS gels.

Work by Zhang and Chandra^13^ showed that recombinant ANCL CSPα mutants formed high molecular weight oligomers *in vitro* in the absence of palmitoylation. At this stage it is not clear if these aggregates that form *in vitro* are the same as those that form in cells. Indeed, mutant aggregates formed within cells are membrane-associated^6^, and as membrane association of CSPα is mediated by palmitoylation, this implies that the aggregates are palmitoylated. Whereas wild-type CSPα has a tendency to form high molecular weight oligomers *in vitro* (in a non-palmitoylated state)^14^, self-association of the wild-type protein in cells is generally limited to the formation of dimers, again highlighting potential differences in the aggregates that form in cells versus *in vitro.* The issue of whether palmitoylation plays a role in promoting/stabilising ANCL CSPα mutant aggregates was questioned by the results of Zhang and Chandra^13^ who did not see any effect of hydroxylamine treatment on aggregation, in contrast to previous work from our group^6^. However, it appears that the conditions used by Zhang and Chandra for hydroxylamine treatment were not sufficient to promote full depalmitoylation of wild-type CSPα and so it is unclear whether these conditions would support the effective depalmitoylation of tightly packed ANCL CSPα mutant aggregates. Besides, the effect of zDHHC enzyme co-expression on aggregation provides clear evidence of the importance of palmitoylation for this process^6^.

Early-onset infantile NCL is caused by mutations in the gene encoding PPT1, an enzyme mediating protein depalmitoylation during degradation in lysosomes. It is fascinating that Henderson *et al.* ^15^ reported a massive increase in PPT1 expression levels in brains of patients carrying the CSPα ANCL mutations. This observation is clearly interesting when considered with our previous proposal that ANCL is associated with accumulation of palmitoylated CSPα aggregates that are resistant to PPT1 action^6^. It will be important in future work to determine the extent of aggregation of ANCL CSPα mutants in brain, as although aggregates are clearly present^6,15^, it is likely that epitope masking prevents an accurate assessment of aggregate levels.

## Methods

### Plasmids

The EGFP-tagged human CSPα (wild type, L115R and ΔL116) plasmid vectors were previously described^6^. Site-directed mutagenesis using oligonucleotide primers (synthesised by Sigma-Aldrich) was used to introduce mutations into the coding sequence of these constructs resulting in substitution of specific cysteines in the encoded proteins. HA-zDHHC3 plasmid was provided by Masaki Fukata^19^.

### Antibodies

Mouse monoclonal GFP antibody (JL8) was purchased from Clontech (California, USA). Anti-HA (rat monoclonal) was purchased from Roche (West Sussex, UK). The rabbit polyclonal CSPα antibody was obtained from Enzo Life Sciences (Exeter, UK). These antibodies were used at dilutions of 1:3,000, 1:1,000 and 1:3,000, respectively for immunoblotting.

### Cell culture, transfection and treatment

Rat pheochromocytoma-12 (PC12) cells were cultured in 75 cm^2^ flasks in RPMI-1640 advanced medium supplemented with 10% horse serum, 5% foetal bovine serum and 1% glutamine. Cells were grown in a humidified atmosphere at 37 °C and 7.5% CO_2_. Human Embryonic Kidney 293 (HEK293T) cells were grown in Dulbecco’s modified Eagle’s media (DMEM) with 10% foetal bovine serum and maintained at a humidified atmosphere of 37 °C and 5% CO_2_. Lipofectamine^TM^2000 reagent (Invitrogen Ltd) was used for all transfections in both PC12 and HEK293T cells following the manufacturer’s instructions.

In order to study the effects of mixing ER and Golgi membranes on the palmitoylation and aggregation of CSPα proteins with C(4-7)L substitutions, transfected PC12 cells were treated for 4 hours with 30 µg/ml of BFA^18^.

### Click chemistry

HEK293T cells were incubated (one day post-transfection) in serum-free DMEM containing 1% (w/v) fatty acid-free bovine serum albumin (BSA) for 30 minutes at 37 °C. Following this, media was removed and cells were incubated in serum-free DMEM supplemented with 1% fatty acid-free BSA containing 100 µM of palmitic acid azide for 4 hours at 37 °C. After metabolic labelling, cells were washed twice in warm PBS and either lysed directly (0 h) or incubated for 3 or 6 hours in serum-free DMEM containing 1% fatty acid-free BSA, containing 100 µM unlabelled palmitic acid and 50 µM of cycloheximide. After this “chase” period, cells were washed twice in ice-cold PBS and lysed on ice with 100 µl of lysis buffer [50 mM Tris (pH 8.0), 0.5% SDS and 1X protease inhibitors]. For the click reaction, 80 µl of click reaction mix (5 μM of alkyne dye, 2 mM of CuSO_4_ and 0.2 mM TBTA in dH_2_O) were added to the cell lysate. After vortexing, 20 µl of ascorbic acid (4 mM) was added, followed by incubation for 1 hour at room temperature with end-over-end rotation. Proteins were precipitated by the addition of three volumes of ice-cold acetone, vortexing and incubation at −20 °C for 20 minutes. Precipitated proteins were pelleted by centrifugation at 15,000 × g for 5 minutes at 4 °C. The samples were washed 3 times in 70% ice-cold acetone and centrifuged each time at 15,000 × g for 5 minutes at 4 °C. The air-dried pellet was resuspended in 100 µl SDS sample buffer containing 25 mM DTT and heated for 5 minutes at 95 °C for analysis by SDS-PAGE.
